# Reliability of crowdsourcing as a method for collecting emotions labels on pictures

**DOI:** 10.1186/s13104-019-4764-4

**Published:** 2019-10-30

**Authors:** Olga Korovina, Marcos Baez, Fabio Casati

**Affiliations:** 1 University of Trento, Trento, Italy; 2 Tomsk Polytechnic University, Tomsk, Russian Federation

**Keywords:** Crowdsourcing emotions, Empirical study, Rating behavior, Reliability

## Abstract

**Objective:**

In this paper we study if and under what conditions crowdsourcing can be used as a reliable method for collecting high-quality emotion labels on pictures. To this end, we run a set of crowdsourcing experiments on the widely used IAPS dataset, using the Self-Assessment Manikin (SAM) emotion collection instrument, in order to rate pictures on valence, arousal and dominance, and explore the consistency of crowdsourced results across multiple runs (reliability) and the level of agreement with the gold labels (quality). In doing so, we explored the impact of targeting populations of different level of reputation (and cost) and collecting varying numbers of ratings per picture.

**Results:**

The results tell us that crowdsourcing can be a reliable method, reaching excellent levels of reliability and agreement with only 3 ratings per picture for valence and 8 per arousal, with only marginal difference between target populations. Results for dominance were very poor, echoing previous studies on the data collection instrument used. We also observed that specific types of content generate diverging opinions in participants (leading to higher variability or multimodal distributions), which remain consistent across pictures of the same theme. These can inform the data collection and exploitation of crowdsourced emotion datasets.

## Introduction

The popularity of digital photography along with the explosion in volume of online social data have greatly motivated and promoted the research on large-scale multimedia analysis and the development of novel concepts for exploiting the expressive nature of images. Doing so requires annotated datasets to guide the analyses and train algorithms. The most widely used datasets in this regard, the International affective picture system (IAPS) dataset [1] and the Geneva affective picture database (GAPED) [2], rely on normative values collected in controlled settings. The need for larger volumes of pictures, however, are motivating researchers to turn to crowdsourcing as an alternative method for collecting emotion labels.

Using crowdsourcing as a method for collecting emotion labels comes however with its challenges. Concerns about running subjective and qualitative studies in uncontrolled remote settings [3] as well as known biases associated to the method [4] require the use and design of crowdsourcing tasks to be carefully planned. This means that unlike data collection in controlled environments, in crowdsourcing we have to deal with strategies of quality control, task design, and proper sampling of participants so as to have reliable results. In this regard, crowdsourced datasets (e.g., [5, 6]) rely on a variety of different techniques (sometimes not fully detailed) in terms of task designs, number of ratings in the dataset generation, and others, leaving researchers with no clear guidelines for how to run such data collection processes.

In this paper we study if and under what conditions crowdsourcing can be used as a reliable method for collecting high-quality emotion labels on pictures. To this end, we run a set of crowdsourcing experiments on a representative set of the IAPS dataset, using the Self-Assessment Manikin (SAM) [7] emotion collection instrument, in order to rate pictures on valence, arousal and dominance.

## Main text

### Methods

#### Research questions

We focus on the following specific research questions:

- RQ1. How reliable is crowdsourcing as a method for collecting emotion labels about pictures? We particularly study *if* and under *what configuration* (e.g., number of collected labels, target population) results can be considered reliable.
- RQ2. How does crowdsourced labels compare to those collected in lab settings? We focus on the results of the process and compare whether crowdsourced labels collected in *uncontrolled* settings can approximate the labels collected in *controlled* lab settings.

#### Dataset

We base our experiments on the widely used IAPS dataset [1]. This dataset contains 1182 images that have been labeled along the three dimensions of *valence*, *arousal*, and *dominance* to indicate emotional reactions. The tags were collected with both paper-and-pencil and computer-based versions of SAM, using a 9-point rating scale for each dimension. The dataset is organized in diverse sets of 60 pictures, each set assigned and annotated in full by 8–25 persons. IAPS was created in the US, but repetitions of the same emotion labeling method in different cultures have resulted in similar results [8]. For our experiments we took a set of 60 pictures, which constitutes a sizeable and representative sample.

#### Experimental design

The task design is based on the instructions and emotion collection methods used in the original IAPS study but adapted to the crowdsourcing environment and ethics [9]. In this sense, and unlike the original IAPS, the annotation task was divided in three pages of 20 pictures each, and workers could decide how many pages to label (min 1 page with 20 pictures). This was to reduce worker’s fatigue and give them more control about when to stop, especially considering the sensitive nature of some of the pictures (e.g., sexual, mutilations). We include screenshots of the task design in Additional file 1.

On top of this task design, we explored two specific dimensions:

- *Target population*, refers to the trade-off between level of reputation of workers, and the availability and costs of worker contributions. We explored two conditions: (i) *specialized* and expensive, focusing on contributors from English-speaking countries belonging to F8 top-tier, as a way of approximating the demographics and level of trust of a controlled lab environment, (ii) *general* and cheaper, focusing on contributors from the rest of the world belonging to F8 s-tier, a much larger pool of workers composed by a cheaper but less reputable population.
- *Number of ratings*, refers to the number of ratings to be collected for each picture in order to reach repeatable results. This aspect was simulated based on the dataset of 30–50 crowdsourced ratings per picture and therefore did not impact the task configuration. To simulate the crowdsourcing runs, we selected random groups of workers so as to have 30 runs per each rating size (1–15 ratings per picture) and computed the mean and standard deviation of the pictures in each run. As a result, we had 30 simulated runs per each rating size.

We run two tasks featuring the specialized (expensive) and the general (cheaper) settings, which amounted to 2cents and 1cent respectively. Each task was running for a week-long period starting September 2019.

#### Metrics

In addressing the research questions, we rely on two main measures:

- *Consistency*. To assess the reliability of crowdsourcing as a method (RQ1), we compute inter-class correlation (two-way random effects, single rater/measurement) between the mean values of multiple crowdsourcing runs. The measure, represented as $ICC_{\text{consistency}}$, focus on the definition of *consistency* which denotes whether participants’ scores to the same group of pictures are correlated in an additive manner [10].
- *Agreement*. When comparing the resulting crowdsourced labels with the gold labels from IAPS (RQ2), we compute the inter-class correlation, $ICC_{\text{agreement}}$ but focusing on the agreement definition which in this case relates to the absolute agreement between the methods.

### Results

#### Reliability of crowdsourcing for emotion labelling

**Fig. 1:**
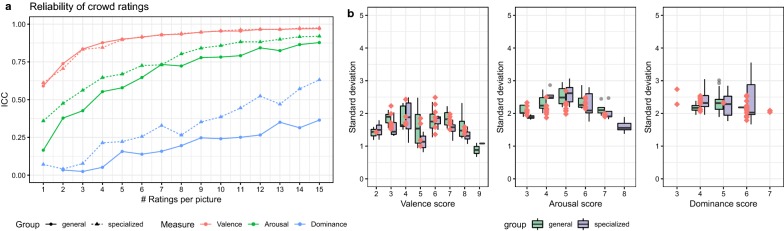
**a** Reliability of crowd ratings per metric and group. **b** Distribution of standard deviation per emotion dimension score

The experimental results summarized in Fig. 1a show that crowdsourcing can be a reliable method for assessing *valence*, reaching an excellent level of consistency (${\text{ICC}}_{\text{consistency}} > . 7 5$) with only 3 ratings per picture. Collecting emotion labels for the *arousal* dimension can also reach similar level of agreement but with at least 8 ratings per picture. *Dominance* showed a lot of variability between runs, achieving fair consistency levels (${\text{ICC}}_{\text{consistency}} > . 4$) only with a large number of ratings. Comparing the target populations, we see not surprisingly that the *specialized* group performed more reliably than the *general* population. Interestingly, however, the performance is comparable for valence, slightly lower for arousal (around 1 rating difference) and more noticeable only for dominance.

Digging into the variability, we observed that the standard deviation in the ratings tend to be higher around the neutral range of the scale and lower at the extremes, suggesting a lower variability in the most extreme emotions (see Fig. 1b).

#### Characterizing the distribution of crowd votes

**Fig. 2:**
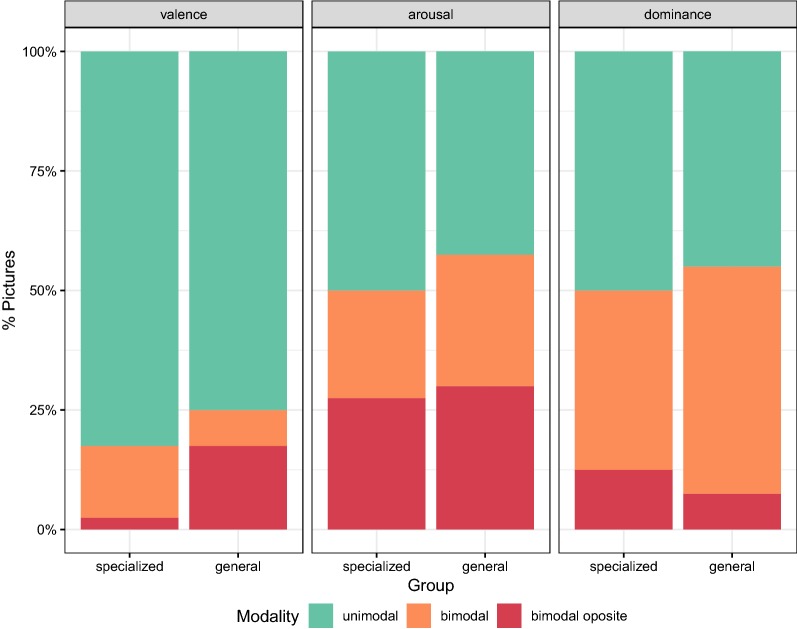
Distribution modality by group and metric

The original IAPS dataset relied on mean and standard deviation to aggregate and report the results. Considering the heterogeneity of the crowd population, we examine the distribution of ratings to assess whether this is a reasonable choice for crowdsourced labels (see Fig. 2).

Looking into the modality of crowd ratings per picture (computed using the dip test [11]) we see that for valence around 75% of the pictures featured a *unimodal* distribution, while for arousal and dominance between 40 and 50%, suggesting that groups perceived pictures more differently for the latter dimensions. Polarizing views were particularly found in arousal. Examples of pictures triggering opposite reactions in terms of arousal are P4609 (couple in a sensual pose) and P9470 (demolished building), which arose calm or excited reactions among participants in both target populations.

Given the above results, we investigated whether there is consistency to the diverging opinions in subgroups of participants, and if those opinions can be transferred from one type of picture to another. To this end, we took pairs of multimodal pictures on the arousal dimension, transformed them to a binary scale and looked at the number of participants who rated both pictures below (0-0) and above (1-1) the neutral value, and those who switched ratings. The results (included in Additional file 2) tell us that people tend to maintain their opinions across pictures featuring themes that relate to their sensitivity (violence, accidents, mutilations), preferences or affinities (sport, children). Thus, depending on the type of content, people might show more or less diverging opinions.

#### Quality of collected labels

**Fig. 3:**
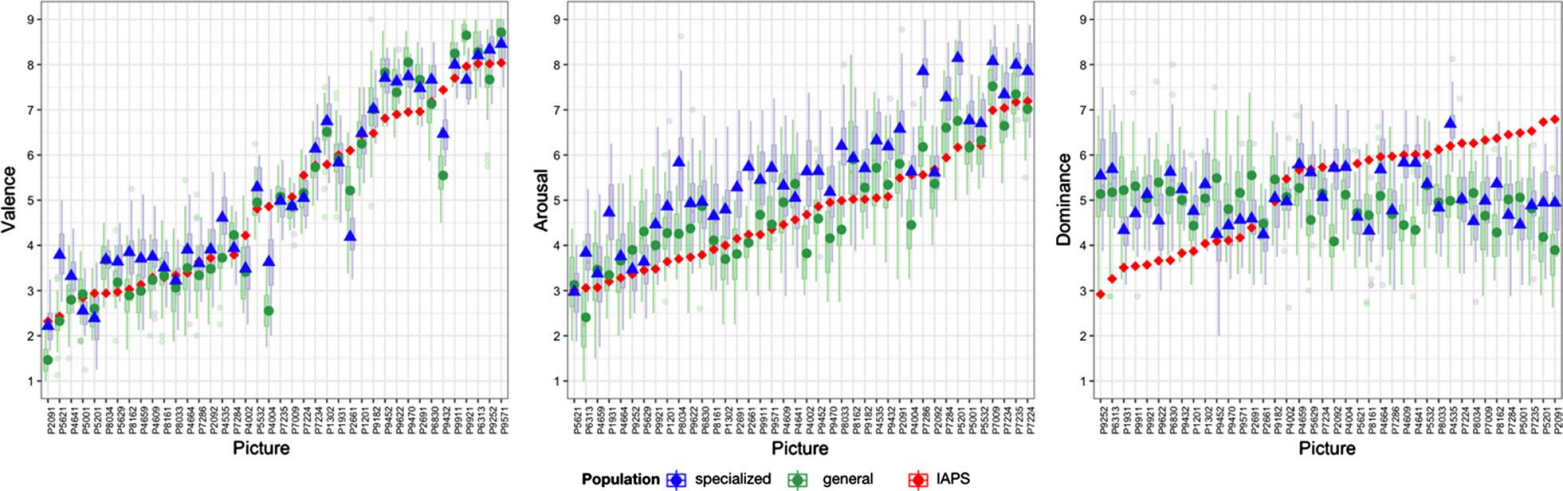
Distribution of crowd votes per picture and group, along with the mean values from the IAPS dataset

In order to compare the quality of crowdsourced emotion labels to those of controlled lab settings, we took the mean ratings from the simulated runs with 8 ratings (same number of ratings in the original dataset) and compared it to those reported in IAPS (see Fig. 3).

The results show crowdsourced labels for *valence* to be of high quality, as the resulting labels feature very high levels of agreement (${\text{ICC}}_{\text{agreement}} > . 9 4$) with IAPS. The few notable deviations can be seen in pictures P4004 (woman posing with her breast), P9434 (breast cancer survivor) where both crowd groups rated more negatively (especially the general population), and P2661 (premature baby taking a bath) where the specialized population rated lower. These deviations can be attributed to different demographic populations and potential cultural perceptions.

Results for *arousal* also show high levels of consistency and agreement between the crowdsourced and original labels from IAPS. Breaking down the results by group, we see that while the specialized group shows slightly higher consistency with IAPS (${\text{ICC}}_{\text{agreement}} > . 9 1 1$, ${\text{ICC}}_{\text{agreement}} > . 7 4 1$) than the general population (${\text{ICC}}_{\text{agreement}} > . 8 9 3$, (${\text{ICC}}_{\text{agreement}} > . 8 9 2$), he latter features a higher absolute agreement. The reason as observed in Fig. 3 is that the specialized group consistently displayed higher levels of arousal than IAPS (pairwise comparison with bonferroni correction showing a significant difference between the mean values at p = .007), while the general group concentrated its values around the original labels. Crowdsourcing the dominance dimension results in low-quality labels.

We also compared the impact of (specialized crowd) workers rating all pictures as opposed to batches of 20. The results were very similar for valence (${\text{ICC}}_{\text{agreement}} = . 9 1 5$), significantly dropped for arousal (${\text{ICC}}_{\text{agreement}} > . 4 3 3$) and improved for dominance (${\text{ICC}}_{\text{agreement}} = . 6 7 5$) although still moderate. This insight calls for further investigation into the tradeoff between worker variability and potential fatigue in large scale emotion collection.

## Discussion

Crowdsourcing can be a reliable method for collecting high-quality emotion labels, for valence and arousal (3–8 ratings) but not for dominance. The results for dominance are in lines with previous empirical studies with IAPS as stimuli dataset and SAM as affect measurement instrument. Bradley and Lang [12], in their seminal study observed dominance to be the dimension with higher variability in terms of semantic label. They concluded that SAM might be better suited for capturing the individual’s and not the stimulus feelings of control. Given the diversity of crowdsourcing workers, this can be one contributing factor. Another explanation relates to SAM requiring additional instructions to support the pictorial representations and the dominance dimension being more difficult to explain [13]. Thus, an uncontrolled experiment without the researcher to clarify doubts could lead to higher variability.

The type of pictures can lead to diverging opinions, which tend to be consistent and transferable to pictures of similar themes. We noticed that some variability in the ratings can be expected in (i) more neutral pictures—where the differences in the mean tend to be higher than pictures depicting stronger emotions—and (ii) depending on the content of the pictures, especially around themes that are more sensible to demographics and cultural settings. This was particularly true for the arousal and dominance dimensions—although for the latter, for the reasons addressed before. In this regard, validations of the IAPS dataset across countries have also shown different rating scores for arousal for the same set of pictures (e.g., [14, 15]). This, along with gender differences, stresses the importance of carefully selecting the target crowd.

### Limitations

While the results can potentially be generalized to other types of emotion collection instruments, the empirical results are limited to SAM and pictures with the level of subjectivity found in IAPS.

## Supplementary information

**Additional file 1.** Task design for collecting crowdsourced labels.

**Additional file 2.** Rating behavior across multimodal pictures for arousal. The 0-0 and 1-1 columns denote the consistency in rating below and above the neutral value. The column “change” indicate those who switched their rating. Values indicate number of participants.

## Data Availability

The datasets used and/or analyzed during the current study are available from the corresponding author on reasonable request.

## Abbreviations

SAM: Self-Assessment Manikin
IAPS: International Affective Picture System
