# Supplementary material for: Reliability of crowdsourcing as a method for collecting emotions labels on pictures
Source: BMC Res Notes. 2019 Oct 30;12:715. doi: 10.1186/s13104-019-4764-4 (PMC6822440; doi:10.1186/s13104-019-4764-4)
Supplement: Supplementary file 1 — Additional file 1. Task design for collecting crowdsourced labels. [file 13104_2019_4764_MOESM1_ESM.pdf]

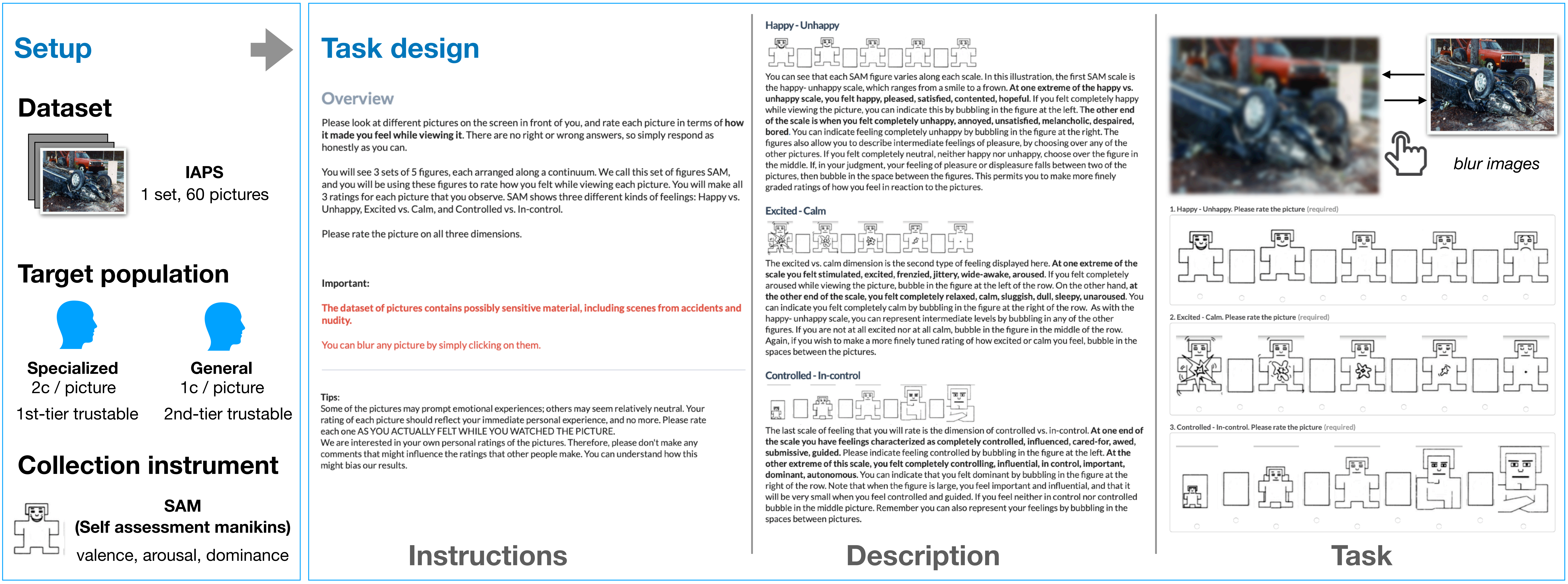

Setup
Dataset
IAPS
1 set, 60 pictures
Target population
Specialized
2c / picture
1st-tier trustable
General
1c / picture
2nd-tier trustable
Collection instrument
SAM
(Self assessment manikins)
valence, arousal, dominance
Task design
Overview
Please look at different pictures on the screen in front of you, and rate each picture in terms of how it made you feel while viewing it. There are no right or wrong answers, so simply respond as honestly as you can.
You will see 3 sets of 5 figures, each arranged along a continuum. We call this set of figures SAM, and you will be using these figures to rate how you felt while viewing each picture. You will make all 3 ratings for each picture that you observe. SAM shows three different kinds of feelings: Happy vs. Unhappy, Excited vs. Calm, and Controlled vs. In-control.
Please rate the picture on all three dimensions.
Important:
The dataset of pictures contains possibly sensitive material, including scenes from accidents and nudity.
You can blur any picture by simply clicking on them.
Tips:
Some of the pictures may prompt emotional experiences; others may seem relatively neutral. Your rating of each picture should reflect your immediate personal experience, and no more. Please rate each one AS YOU ACTUALLY FELT WHILE YOU WATCHED THE PICTURE.
We are interested in your own personal ratings of the pictures. Therefore, please don't make any comments that might influence the ratings that other people make. You can understand how this might bias our results.
Instructions
Happy - Unhappy
You can see that each SAM figure varies along each scale. In this illustration, the first SAM scale is the happy- unhappy scale, which ranges from a smile to a frown. At one extreme of the happy vs. unhappy scale, you felt happy, pleased, satisfied, contented, hopeful. If you felt completely happy while viewing the picture, you can indicate this by bubbling in the figure at the left. The other end of the scale is when you felt completely unhappy, annoyed, unsatisfied, melancholic, despaired, bored. You can indicate feeling completely unhappy by bubbling in the figure at the right. The figures also allow you to describe intermediate feelings of pleasure, by choosing over any of the other pictures. If you felt completely neutral, neither happy nor unhappy, choose over the figure in the middle. If, in your judgment, your feeling of pleasure or displeasure falls between two of the pictures, then bubble in the space between the figures. This permits you to make more finely graded ratings of how you feel in reaction to the pictures.
Excited - Calm
The excited vs. calm dimension is the second type of feeling displayed here. At one extreme of the scale you felt stimulated, excited, frenzied, jittery, wide-awake, aroused. If you felt completely aroused while viewing the picture, bubble in the figure at the left of the row. On the other hand, at the other end of the scale, you felt completely relaxed, calm, sluggish, dull, sleepy, unaroused. You can indicate you felt completely calm by bubbling in the figure at the right of the row. As with the happy- unhappy scale, you can represent intermediate levels by bubbling in any of the other figures. If you are not at all excited nor at all calm, bubble in the figure in the middle of the row. Again, if you wish to make a more finely tuned rating of how excited or calm you feel, bubble in the spaces between the pictures.
Controlled - In-control
The last scale of feeling that you will rate is the dimension of controlled vs. in-control. At one end of the scale you have feelings characterized as completely controlled, influenced, cared-for, awed, submissive, guided. Please indicate feeling controlled by bubbling in the figure at the left. At the other extreme of this scale, you felt completely controlling, influential, in control, important, dominant, autonomous. You can indicate that you felt dominant by bubbling in the figure at the right of the row. Note that when the figure is large, you feel important and influential, and that it will be very small when you feel controlled and guided. If you feel neither in control nor controlled bubble in the middle picture. Remember you can also represent your feelings by bubbling in the spaces between pictures.
Description
blur images
1. Happy - Unhappy. Please rate the picture (required)
2. Excited - Calm. Please rate the picture (required)
3. Controlled - In-control. Please rate the picture (required)
Task
