# Supplementary material for: Reliability of crowdsourcing as a method for collecting emotions labels on pictures
Source: BMC Res Notes. 2019 Oct 30;12:715. doi: 10.1186/s13104-019-4764-4 (PMC6822440; doi:10.1186/s13104-019-4764-4)
Supplement: Supplementary file 2 — Additional file 2. Rating behavior across multimodal pictures for arousal. The 0-0 and 1-1 columns denote the consistency in rating below and above the neutral value. The column “change” indicate those who switched their rating. Values indicate number of participants. [file 13104_2019_4764_MOESM2_ESM.pdf]

**Pictures**		**Labels**		**0-0**	**1-1**	**Change** (%)
Specialized						
P9622	P6830	violence	accident	12	15	3 (.100)
P9921	P9622	accident	grotesque	11	14	4 (.138)
P9921	P6830	violence	grotesque	10	14	5 (.172)
P9571	P9470	accident	grotesque	10	14	6 (.200)
P8161	P8034	sport	sport	8	15	6 (.207)
P9911	P9452	assault	accident	8	15	7 (.233)
P9571	P9452	assault	grotesque	7	15	8 (.267)
P9622	P8034	sport	accident	8	14	8 (.267)
P8034	P2091	children	sport	5	16	8 (.276)
General						
P9470	P2691	violence	accident	21	17	10 (.208)
P9622	P9452	assault	accident	15	21	11 (.234)
P9921	P9571	grotesque	grotesque	18	15	12 (.267)
P9452	P9182	grotesque	assault	12	21	13 (.283)
P9622	P9182	grotesque	accident	14	21	14 (.286)
P9252	P2691	violence	grotesque	21	13	14 (.292)
P9470	P9252	grotesque	accident	20	14	14 (.292)
